# The Hospital Officer's Bookshelf

**Published:** 1912-11-16

**Authors:** 


### THE HOSPITAL OFFICER'S BOOKSHELF.

Medical Benefit in Germany and Denmark. By I. G. Gibbon, B.A., D.Sc. (London: P. S. King and Son, Orchard House, Westminster. 1912. Price 6s. net.)

Dr. Gibbon reviews, in a short and concise volume, the whole aspect of medical benefit in Germany and Denmark under the provisions of the local Insurance Acts. Such a theme demanded for its adequate treatment wide reading and perhaps personal experience, of neither of which the author gives sufficient proof. On the other hand, he shows that he has a fair purview of the essentials, and that, after all, is what is demanded for a popular book on the subject. We are too grateful to him and the publishers for having provided a summary of facts which the doctor and layman, unacquainted with the instructive manuals on the same subject which exist in Germany, may read to cavil at his omissions, and we cordially welcome his work as affording an instructive insight, without any attempt at being exhaustive, into the conditions under which medical men in Germany administer the medical service under the Insurance Act. We shall have occasion to return to some of the points he raises; for the present it suffices to give a general outline of the contents and scope of the book, which is one heartily to be recommended to the doctor and the society secretary.

The first two chapters are introductory, and outline the scheme of insurance, with special reference to the medical benefits in Germany and Denmark. The third deals more fully and particularly with medical benefit, while the three succeeding articles treat of the free choice of doctor, and discuss this important point from all its bearings, the author's conclusion being that free choice of doctor is indispensable. Chapters vii. to ix. deal with the question of payment, and Dr. Gibbon urges strongly that adequate payment of the doctors is necessary in the interests of all—a conclusion we heartily endorse. Chapters x. to xii. deal with the vexed question of control of the medical service: the general trend of the writer's conclusions is that strict control is necessary over the doctors when there is free choice of doctor, but that control in expert matters should be mainly in the hands of the profession itself. This matter is undoubtedly a very difficult one to discuss, and the author deals fairly and impartially with both sides, losing thereby nothing of the force which his arguments possess. The thirteenth, fourteenth, and fifteenth chapters treat of medical and surgical requirements; the following three chapters explain the working of the institutional benefits, while the penultimate article is devoted to a discussion of the relations between insurance and the public health authorities. The final chapter summarises the main conclusions to be drawn from this review. Not the least important part of the book are the instructive appendices which give translations of agreements, and deal with particular questions of policy. The book is an able summary, in popular language and simple readable style, of a subject which is difficult enough, and about which comparatively little is known in this country. As such it should appeal to many readers, especially as it comes at a time when medical benefit under the Insurance Act is the burning professional topic of the hour.

